# What are the factors affecting primary care choice when the access under health insurance scheme is limited?: a cross-sectional study in Bandung, Indonesia

**DOI:** 10.1186/s12875-024-02296-6

**Published:** 2024-02-21

**Authors:** Afina Nur Fauziyyah, Akira Shibanuma, Ken Ing Cherng Ong, Masamine Jimba

**Affiliations:** 1 Department of Community and Global Health, The University of Tokyo, Tokyo, Japan; 2 Department of Practice and Policy, School of Pharmacy, University College London, London, United Kingdom

**Keywords:** Primary care, Health insurance, Preference, Choice, Spatial network analysis

## Abstract

**Background:**

Ensuring equal access to primary care services is crucial, as the gateway to a higher level of care. Indonesia has been trying to increase financial access to medical care by administering national health insurance known as BPJS-Health (*Badan Penyelenggara Jaminan Sosial Kesehatan*) since 2014. However, BPJS-Health beneficiaries can only use their benefits at a limited number of registered primary care providers (BPJS-Health partners). This study investigated the geographical coverage of BPJS-Health and BPJS-Health beneficiaries’ primary care choices, based on their characteristics and healthcare preferences in the target areas of Bandung, Indonesia.

**Methods:**

The setting of this cross-sectional study was the areas with low physical access to BPJS-Health partners but high physical access to non-BPJS-Health partners. Physical access was determined by spatial network analysis, resulting in a geographical coverage map. A total of 216 adults were recruited and they completed the questionnaire about their primary care choice. All participants had been registered with the BPJS-Health system and living in the study areas. Their participation in non-BPJS-Health was also evaluated. Participants’ choice of care was assessed in three different scenarios, when the individual was experiencing mild, chronic, and serious illnesses.

**Results:**

BPJS-Health partners’ geographical coverage was unequally distributed in Bandung. Being registered with non-BPJS-Health company was negatively associated with the more frequent choice of using BPJS-Health partners’ services (AOR = 0.18; 95% CI, 0.06-0.58, *P* = 0.004) among BPJS-Health beneficiaries. For serious illnesses, having a high income was associated with choosing non-BPJS-Health partners and hospitals (AOR = 4.90; 95% CI, 1.16-20.77, *P* = 0.031). When dealing with mild and chronic illnesses, participants were concerned about the quality of treatment they would receive as a major factor in choosing a primary care provider. However, receiving better treatment quality was negatively associated with choosing BPJS-Health partners in all cases of illness severities.

**Conclusions:**

Sociodemographic characteristics, healthcare preference factors, and health insurance status were associated with participants’ primary care choices in the target areas of Bandung, Indonesia. BPJS-Health partners’ coverage map and the preference factors are potentially important for policymakers, especially for the development of future BPJS-Health partnerships.

**Supplementary Information:**

The online version contains supplementary material available at 10.1186/s12875-024-02296-6.

## Background

Access to primary care services is one of the core dimensions of a strong health system performance and the distribution of health in the population [1]. Yet, inequities still exist in access to healthcare services in many low and middle-income countries (LMICs) [2]. Since the release of the 2010 World Health Report, efforts have been made to achieve universal health coverage by reducing financial barriers in LMICs [3]. In Indonesia, *Badan Penyelenggara Jaminan Sosial Kesehatan* (Social Security Agency for Health, abbreviated as BPJS-Health) has been administering the national health insurance scheme to increase access to healthcare services.

Under the BPJS-Health system, primary care providers play a central role as the gatekeepers of higher levels of care. To obtain medical cost coverage, BPJS-Health beneficiaries must visit BPJS-Health partners comprised of government primary health centres (*Pusat Kesehatan Masyarakat,* abbreviated as *puskesmas*) and some registered private clinics [4]. If a patient’s illness is outside of the 155 illnesses that cannot be treated at the primary care level, they will receive a referral letter for a higher level of care [4, 5]. BPJS-Health beneficiaries can not use BPJS-Health benefits if they receive medical treatment from non-BPJS-Health partners or hospitals without a referral letter (except in emergencies).

According to the 2017 BPJS-Health report, long waiting time was the most common complaint among BPJS-Health beneficiaries. From the perspective of BPJS-Health partners, the distribution of patients is one of the biggest problems in managing the BPJS-Health system [6]. The availability of BPJS-Health partners’ services still can not meet the need. In the Special Capital Region of Jakarta, the capital city of Indonesia, 7 to 8 *puskesmas* are available per district. However, in West Papua province, the ratio of *puskesmas* is only 0.2 per district [7].

Efforts have been made to solve the distribution problem of BPJS-Health partners. Between 2016 and 2017, 1055 BPJS-Health partners were registered in the system [8, 9]. The BPJS-Health providers continue to increase its partnerships with private primary care providers to cover the growing population. In addition, BPJS-Health will redistribute beneficiaries equally to the nearest primary care provider from the patient’s domicile [6]. However, no information is available on all primary care providers’ physical catchment areas. When increasing the number of partnerships, BPJS-Health beneficiaries’ needs in different regions should be considered, to ensure that the solution is effective for all beneficiaries.

Knowing patients’ preferences can help policymakers prioritize healthcare services that meet patients’ needs [10]. Few studies have assessed the factors that affect the decision to choose primary care services under the BPJS-Health system. In this study, we selected geographical areas where the number of accessible BPJS-Health partners was limited, but there were some non-BPJS-Health partners for the population to use nearby in Bandung. We assessed BPJS-Health geographical coverage and the BPJS-Health beneficiaries’ primary care choices –based on their characteristics and healthcare preferences– in these areas to support the BPJS-Health plan for future partnerships.

## Methods

### Study design and site

We conducted this cross-sectional study in Bandung City, the capital city of West Java province, Indonesia. The data collection period was from September to October 2018. Bandung City has 203 primary care providers (as of August 2018), 109 secondary care providers, and 35 tertiary care providers or hospitals. In this study, we focused on the primary care providers.

### Study procedure and sampling area

We used spatial analysis and a household-based survey to assess the factors associated with Bandung City residents’ preferences in choosing primary care providers. First, we used a spatial analysis to determine the sampling area in which the household-based survey was conducted. The sampling area had the following conditions: the areas with low physical access to BPJS-Health partners but with high physical access to non-BPJS-Health partners. Second, we conducted a household-based survey to determine study participants’ preferences for primary care providers.

### Spatial network analysis to determine the sampling area

**Fig. 1:**
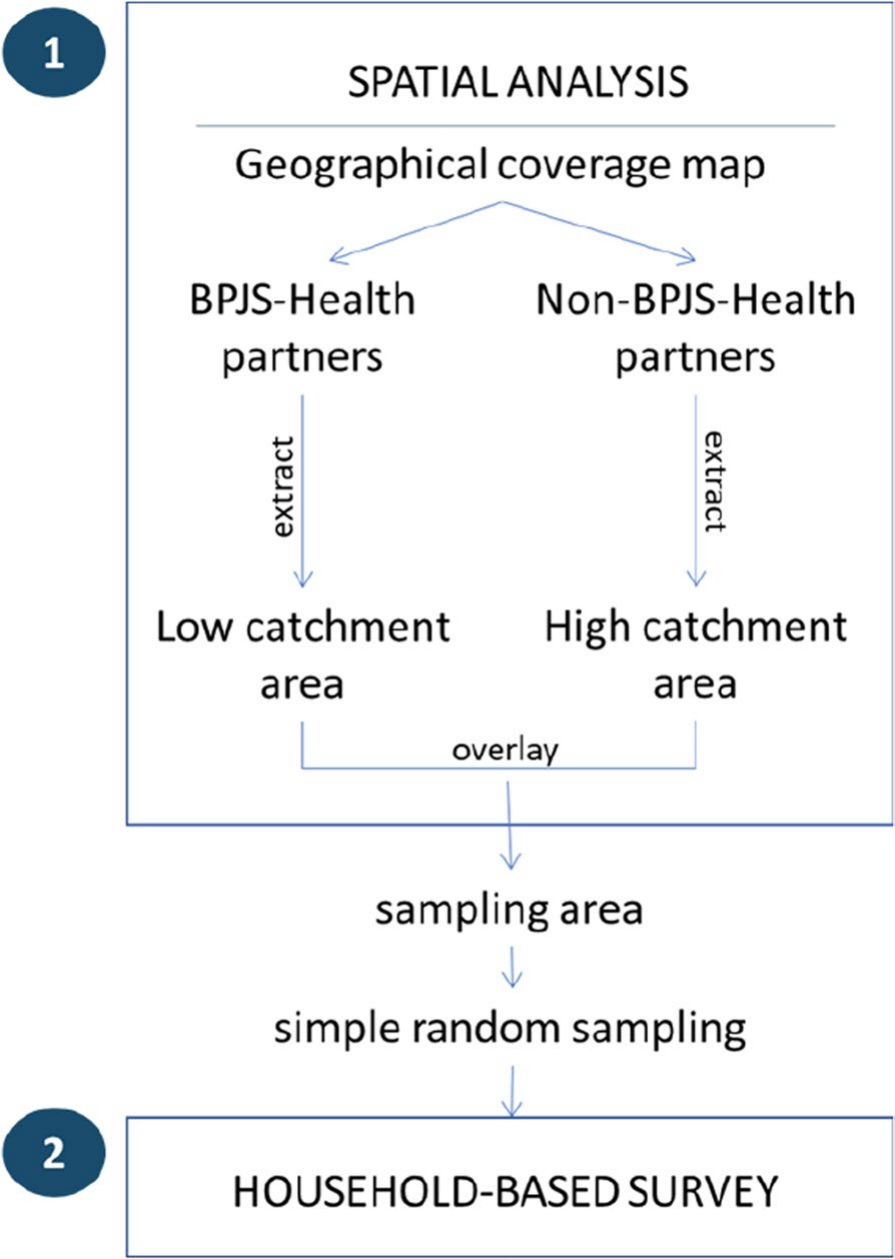
Overview of the study process. BPJS-Health partners: Primary care providers in partnership with *Badan Penyelenggara Jaminan Sosial Kesehatan* (Indonesian National Health Insurance provider)

To determine physical accessibility to BPJS-Health and non-BPJS-Health partners, we created two different spatial coverage maps for primary care; the first was a geographical coverage map of the BPJS-Health partners in Bandung, while the second indicated non-BPJS-Health partners. A total of 106 BPJS-Health partners and 97 non-BPJS-Health partners were included in the spatial analysis. Of those BPJS-Health partners, 49 were *puskesmas* and 57 were private primary care providers. For each map, the first step was to determine the catchment area of each primary care provider point using a network buffering analysis [11]. The Indonesian Ministry of Health reported that the longest travel time to the nearest primary care provider should be 15 minutes by any means [12]. According to the report, most residents (68.7%) used motorcycles to visit primary care providers [12]. The catchment area was set a travel time of 15 minutes by road from the primary care provider. Each catchment area of the primary care providers was valued at 1 (weighed in balance; Fig. 1).

To select the residents, we selected the households based on a simple random sampling method using a random selection tool for ArcGIS software [13]. After that, we extracted the point location (lot-land) of each selected household polygon and opened it in Google My Map to find the address for the household survey. If the selected address could not be reached at the time of the survey (e.g. empty household), the sampling point was changed to the nearest address around the original point. We recorded the actual sampling points in Google My Map to ensure that the points were inside the sampling areas.

### Study participants

The participants of this study were Bandung residents living in the sampling areas. We interviewed the head of the household on behalf of other family members. If a head of the household was not present at the time of the survey, we interviewed other family members who met the following inclusion criteria: (1) Bandung residents (both permanent and temporary residents who lived more than 1 year were included), (2) aged 18 years or older, and (3) registered as BPJS-Health beneficiary. We calculated the sample size (80% power, 95% confidence interval) based on a percentage of health insurance usage for outpatients in West Java province with a percentage of 38.5% [14] . We recruited 30 residents for the pre-test survey. A total of 216 residents (not including pre-test residents) participated in this study, and this study included all of the interviewed residents in the analysis.

### Data collection

We collected secondary datasets for spatial analysis before the household-based survey. Datasets of the Bandung City (road networks, household, and primary care providers plane figure) were available online from the Indonesia Geospatial Portal. The Department of Health of Bandung City was also providing primary care provider's address to validate the primary care provider's point location. We obtained the speed rate for each type of road in Bandung City from OpenStreetMap, an open-source world map. For the household-based survey, the lead researcher and four trained interviewers visited residents’ households to conduct the interview. We interviewed the residents individually for 20-30 minutes.

We conducted the face-to-face interviews using a structured questionnaire. We developed the questionnaire based on a previous study in China and National Welfare Survey 2017 in Indonesia [14, 15] [see Additional file 1]. The dependent variable was the primary care services choice of the resident. The independent variables were sociodemographic characteristics, previous healthcare utilization, and preferences of healthcare [10, 14–20]. Using the questionnaire, we assessed residents’ choice of care in different conditions: most of the time, and in hypothetical situations of having mild, chronic, and serious illnesses [21]. Mild and chronic illnesses in this study were the illnesses that should be able to be treated in a primary care setting, listed in 155 common illnesses [5]. We also asked about the residents’ status of BPJS-Health, whether registered as PBI (*Penerima Bantuan Iuran*)—those who were fully covered by the government—or non-PBI, those who had to pay the premium each month. We pretested the questionnaire among 30 residents living inside the sampling areas in advance. After the pretest, we received feedback from them, but no change was made to the questionnaire. We then assessed whether the 216 residents would self-medicate, or seek care from medical personnel in the pharmacy, BPJS-Health partners, or non-BPJS-Health partners.

### Data analysis

We conducted a spatial analysis using ArcGIS version 10.2 (Redlands, California) and statistical analysis using Stata version 13.1 (College Station, Texas). We excluded data with missing information from the analysis. We used chi-square and independent samples t-test to compare the difference in residents’ general characteristics between frequent BPJS-Health and non BPJS-Health partners’ users. We conducted principal component analysis (PCA) to elicit major preference factors in choosing a primary care provider. In PCA analysis, we set variable loadings cut-off as 0.32 and included all principal components with eigenvalue >1.00 to the logistic regression analysis. By multiple logistic regression and multinomial logistic regression, we addressed factors associated with residents’ primary care choice by residents’ characteristics and major preference factors. The statistical significance level was set at 5%.

### Ethical consideration

We obtained approval from the Research Ethics Committee of the Graduate School of Medicine, the University of Tokyo, Japan (2018022NI), and the Research and Community Engagement Ethical Committee of the Faculty of Public Health at the University of Indonesia (777/UN2.F10/PPM.00.02/2018). We also obtained formal permissions from the National Unity, Politics, and Community Protection Agency of Indonesia and the Department of Health of Bandung City. Participation was voluntary and the residents could withdraw from the study at any time. They gave written informed consent after a short explanation about the study. We analyzed all data anonymously and maintained the confidentiality of the entire data set strictly.

## Results

### Spatial analysis results, sociodemographic characteristics, and residents’ health service utilization

**Fig. 2:**
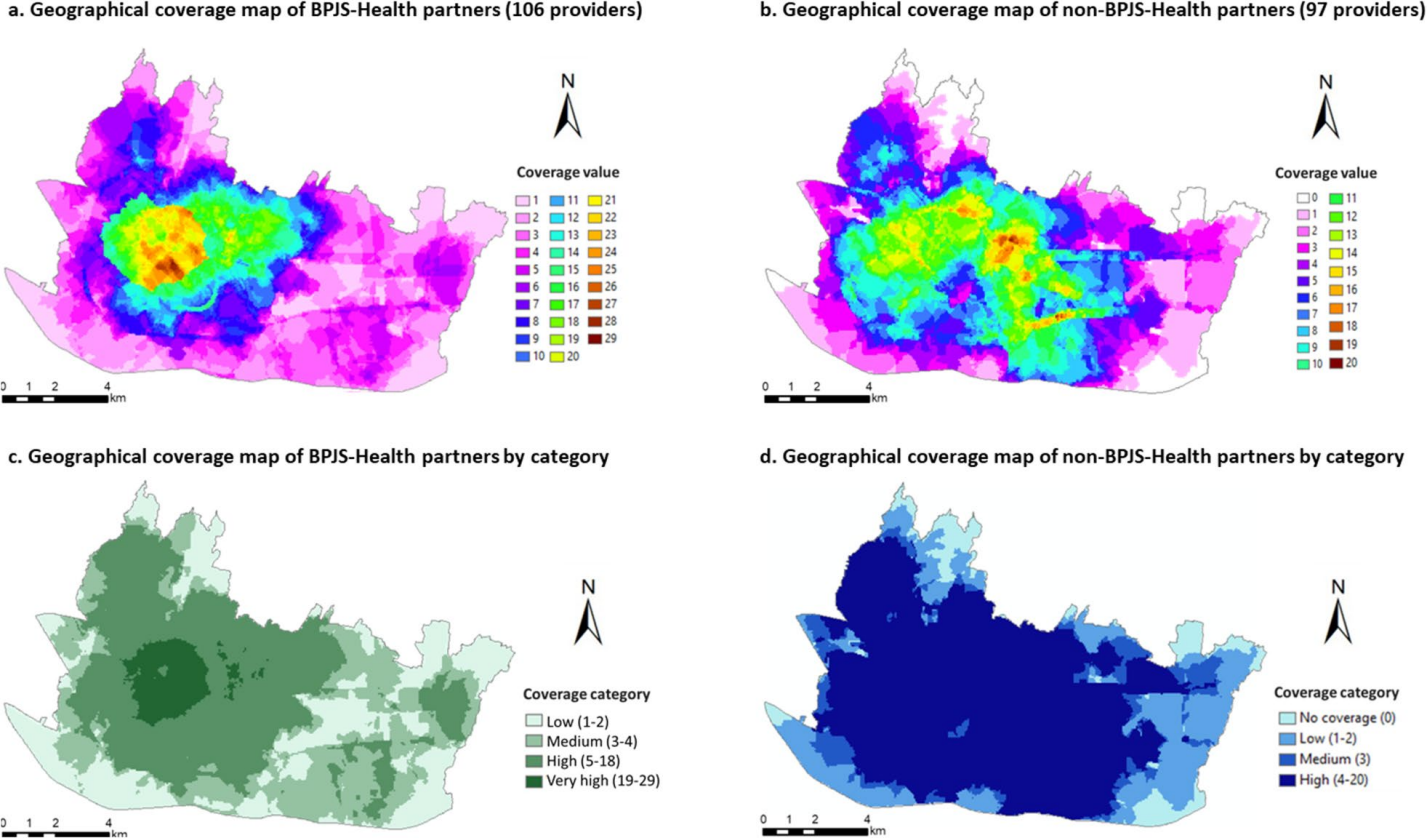
Geographical coverage map of BPJS-Health and non-BPJS-Health partners. BPJS-Health partners : Primary care providers in partnership with BPJS-Health (*Badan Penyelenggara Jaminan Sosial Kesehatan*–Indonesian National Health Insurance)

**Fig. 3:**
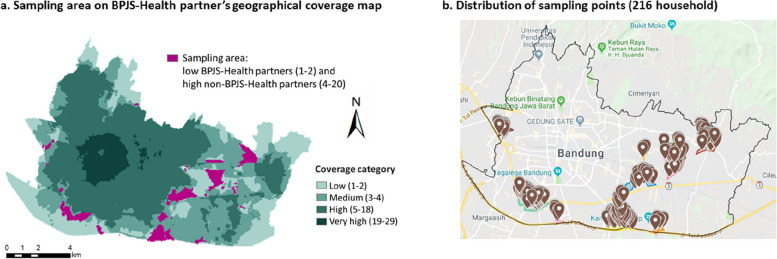
Sampling areas for household-based survey. BPJS-Health partners : Primary care providers in partnership with BPJS-Health (*Badan Penyelenggara Jaminan Sosial Kesehatan*–Indonesian National Health Insurance)

Each area in Bandung City had a specific coverage value, based on the number of catchment areas overlayed in that area. We grouped the areas into five categories based on the ratio of primary care providers per district in West Java province [22]. The categories were: areas with very high, high, medium, low, and no coverage. For the BPJS-Health partners coverage map, the coverage level was set as follows: 1 to 2 coverage value for low coverage level, 3 to 4 for medium coverage level, 5 to 18 for high coverage level, and 19 to 29 for very high coverage level. For the non-BPJS-Health partners coverage map, the coverage level was set as follows: 0 for no coverage, 1-2 for low coverage level, 3 for medium coverage level, and 4 to 20 for high coverage level. We selected areas with high coverage values for non-BPJS-Health partners, but low coverage values for BPJS-Health partners as the sampling areas. Figure 2 shows the geographical coverage of the BPJS-Health and non-BPJS-Health partners, and Fig. 3 presents the results for the target sampling areas.

**Fig. 4:**
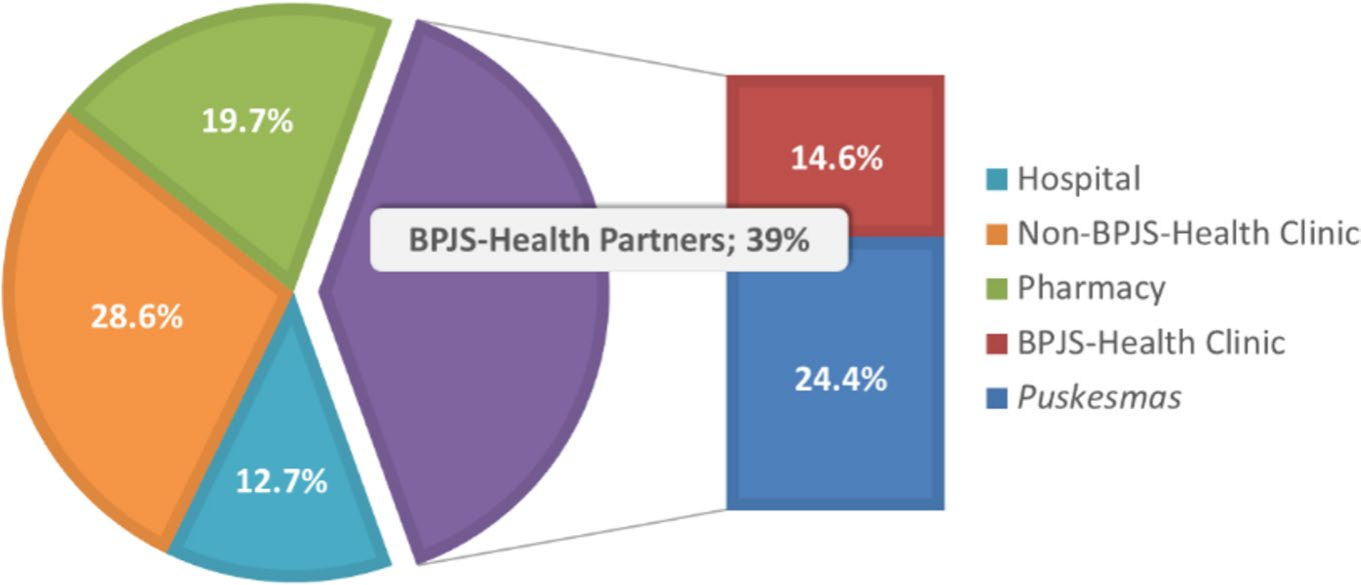
Residents’ health service utilization (*n*=213). BPJS-Health : *Badan Penyelenggara Jaminan Sosial Kesehatan* (Indonesian National Health Insurance)

The sociodemographic characteristics varied among the 216 residents (Table 1). Approximately half (51.4%) of the household heads had a secondary school education. Regarding monthly household income, 116 residents stated that their monthly household income was between Rp 600,000 and Rp 5,000,000 (1 USD = Rp 14,561, as of December 19^th^, 2018). Table 1 also shows residents’ health status and health service utilization. Regarding residents’ choice of health facility, 39% chose BPJS-Health partners as their most frequently visited health facility (Fig. 4). Residents who registered as PBI (*Penerima Bantuan Iuran*)—those who do not have to pay premium or fully covered by the government—were 69.8%. Approximately 20% of the residents were registered with other health insurance providers.

**Table 1:** Sociodemographic characteristics and health service utilization of residents (*n*=216)

**Variable**	**BPJS-Health**		**Non-BPJS-Health**		***p*-value**
	***n***	**%**	***n***	**%**	
Age (range 18-82; mean 44)					0.872
Gender					0.347
Female	54	40.9	78	59.1	
Male	29	34.5	55	65.5	
Head of household occupation					0.542
None	10	40.0	15	60.0	
Entrepreneur	27	37.0	46	63.0	
Civil servant	3	27.3	8	72.7	
Employee	35	41.2	50	58.8	
Freelancer	6	54.5	5	45.5	
Retiree	2	18.2	9	81.8	
Monthly income (Rp) [1USD= Rp 14,561^a^], *n*=211					<0.001
<600,000	19	51.4	18	48.6	
600,000-5,000,000	52	44.8	64	55.2	
>5,000,000	10	17.2	48	82.8	
Head of household education					0.004
Primary school or lower	22	57.9	16	42.1	
Secondary school	44	39.6	67	60.4	
Higher	17	25.4	50	74.6	
Experienced mild illnesses during the past year					0.150
Yes	73	40.6	107	59.4	
No	10	27.8	26	72.2	
Having chronic illnesses					0.112
Yes	31	46.3	36	53.7	
No	52	34.9	97	65.1	
Had hospitalization during the past year					0.446
Yes	13	44.8	16	55.2	
No	70	37.4	117	62.6	
Annual number of consultation with doctor					<0.001
0	14	18.2	63	81.8	
1-3	53	50.5	52	49.5	
>3	16	47.1	18	52.9	
Annual medical cost (Rp) [1USD= Rp 14,561^a^], *n*=214					0.133
<250,000	57	44.2	72	55.8	
250,000-2,000,000	24	32.9	49	67.1	
2,000,000-5,000,000	2	22.2	7	77.8	
>5,000,000	0	0.0	3	100.0	
Medical cost burden, *n*=214					0.585
None	64	40.3	95	59.7	
A bit	13	38.2	21	61.8	
Can not undertake	6	28.6	15	71.4	
Status of BPJS-Health Beneficiary, *n*=215					0.004
PBI	48	32.0	102	68.0	
Non-PBI (Paying premium each month)	35	53.8	30	46.2	
Have ever used BPJS-Health benefit					<0.001
Yes	66	52.8	59	47.2	
No	17	18.7	74	81.3	
Registered to other insurance					<0.001
No	79	44.1	100	55.9	
Yes	4	10.8	33	89.2	

^a^1USD= Rp 14,561 (conversion rate as of December 19th, 2018)

*BPJS-Health* Badan Penyelenggara Jaminan Sosial Kesehatan (Indonesian National Health Insurance), *PBI* Penerima Bantuan Iuran (Full-covered BPJS-Health beneficiaries by government)

### Factors associated with primary care choice by residents’ characteristics

Table 2 presents the factors associated with residents’ utilization of BPJS-Health partners across residents’ characteristics, under different illness conditions. Being a PBI was positively associated with more frequent use of BPJS-Health partners’ services than the non-PBI group (adjusted odds ratio [AOR] = 2.40; 95% CI, 1.02-5.60, *P* = 0.044). However, being registered with other insurance was negatively associated with choosing BPJS-Health partners (AOR = 0.18; 95% CI, 0.06-0.58, *P* = 0.004). For mild illnesses, residents with chronic illnesses (relative risk ratio [RRR] self-medication = 2.99; non-BPJS-Health = 3.68) or those registered to other insurance (RRR self-medication = 4.27; non-BPJS-Health = 4.34) preferred to self-medicate or visit non-BPJS-Health partners, over BPJS-Health partners. Residents with higher education also preferred self-medication for mild illness (RRR = 5.11; 95% CI, 1.22-21.40, *P* = 0.026).

**Table 2:** Factors associated with primary care choice by residents’ characteristics

**Condition**	**Variables**	**Primary care choice (AOR/RRR [95% CI])**		
		**Pharmacy**	**Self-medication**	**Non-BPJS-Health**
Actual choice (*n*=210)	Age (range 18-82; mean 44)			0.97 (0.94-1.00)
	Having chronic illnesses (ref: no)			0.95 (0.45-2.00)
	Monthly income (Rp) [ref: <600,000]^a^			
	600,000-5,000,000			0.94 (0.34-2.62)
	>5,000,000			0.50 (0.12-2.08)
	Head of household education (ref: primary or lower)			
	Secondary			0.63 (0.23-1.69)
	Higher			0.50 (0.16-2.08)
	PBI (ref: non-PBI)			2.40 (1.02-5.60)*
	Registered to other insurance (ref: no)			0.18 (0.06-0.58)**
Mild illnesses (*n*=210)	Age (range 18-82; mean 44)	0.92 (0.85-0.98)*	1.03 (0.99-1.06)	1.03 (0.99-1.08)
	Having chronic illnesses (ref: no)	0.74 (0.11-4.82)	2.99 (1.22-7.29)*	3.68 (1.22-11.08)*
	Monthly income (Rp) [ref: <600,000]^a^			
	600,000-5,000,000	4.24 (0.61-29.48)	0.55 (0.19-1.61)	1.60 (0.23-11.21)
	>5,000,000	5.28 (0.41-67.82)	0.38 (0.08-1.72)	2.81 (0.26-30.59)
	Head of household education (ref: primary or lower)			
	Secondary	0.27 (0.03-2.18)	2.00 (0.61-6.60)	2.34 (0.31-17.81)
	Higher	0.14 (0.01-2.22)	5.11 (1.22-21.40)*	5.13 (0.56-47.36)
	PBI (ref: non-PBI)	0.50 (0.11-2.17)	0.61 (0.24-1.58)	0.48 (0.11-2.10)
	Registered to other insurance (ref: no)	3.16 (0.51-19.58)	4.27 (1.19-15.32)*	4.34 (1.08-17.49)*
Chronic Illnesses (*n*=209)	Age (range 18-82; mean 44)		1.00 (0.97-1.04)	1.01 (0.98-1.03)
	Having chronic illnesses (ref: no)		3.16 (1.08-9.25)*	0.72 (0.33-1.57)
	Monthly income (Rp) [ref: <600,000]^a^			
	600,000-5,000,000		0.72 (0.21-2.43)	1.56 (0.60-4.02)
	>5,000,000		1.12 (0.19-6.45)	2.15 (0.51-9.03)
	Head of household education (ref: primary or lower)			
	Secondary		0.24 (0.06-0.93)*	0.80 (0.28-2.27)
	Higher		0.15 (0.02-0.87)*	1.84 (0.48-7.12)
	PBI (ref: non-PBI)		0.51 (0.16-1.65)	0.99 (0.44-2.22)
	Registered to other insurance (ref: no)		0.52 (0.05-5.23)	4.78 (1.60-14.30)**
Serious Illnesses (*n*=199)	Age (range 18-82; mean 44)			0.99 (0.96-1.03)
	Having chronic illnesses (ref: no)			0.84 (0.39-1.82)
	Monthly income (Rp) [ref: <600,000]^a^			
	600,000-5,000,000			2.43 (0.94-6.31)
	>5,000,000			4.90 (1.16-20.77)*
	Head of household education (ref: primary and lower)			
	Secondary			1.41 (0.49-4.09)
	Higher			1.08 (0.29-3.96)
	PBI (ref: non-PBI)			0.43 (0.18-1.00)*
	Registered to other insurance (ref: no)			3.48 (0.65-18.74)

Reference category: BPJS-Health partners . Adjusted for gender, experienced mild illnesses during the past year, annual number of consultation with doctor, experience of using BPJS-Health benefit

*BPJS-Health* Badan Penyelenggara Jaminan Sosial Kesehatan (Indonesian National Health Insurance), *PBI* Penerima Bantuan Iuran (Full-covered BPJS-Health beneficiaries by government)

^*^p<0.05, **p<0.01, *AOR* Adjusted odds ratio, *RRR* Relative risk ratio, *CI* Confidence intervals

^a^1USD= Rp 14,561 (conversion rate as of December 19th, 2018)

Education, health status, and other insurance membership were factors associated with residents’ primary care choices when suffering from chronic illnesses. Residents with chronic illnesses were more likely to choose self-medication compared to BPJS-Health partners (RRR = 3.16; 95% CI, 1.08-9.25, *P* = 0.036). In contrast, residents preferred not to choose self-medication if they had a higher level of education (RRR secondary=0.24; higher=0.15). On the other hand, being registered with other insurance was positively associated with choosing non-BPJS-Health partners (RRR = 4.78; 95% CI, 1.60-14.30, *P* = 0.005).

When residents suffered from serious illnesses, the monthly household income and BPJS-Health status of beneficiaries affected their choice of healthcare. Having a high monthly income (more than Rp 5,000,000) was positively associated with a lower probability of choosing BPJS-Health partners for serious illnesses (AOR = 4.90; 95% CI, 1.16-20.77, *P* = 0.031). In contrast, being registered as a PBI was positively associated with higher BPJS-Health partner utilization (AOR = 0.43; 95% CI, 0.18-1.00, *P* = 0.05).

### Major preference factors in choosing primary care

The PCA results showed five major preference factors for illness severity (Table 3). The answers from 211 residents’ resulted in five major preference factors that represented 55.7% of the variance in mild illness. The major preference factors were accessibility and acceptability, treatment quality, affordability, physical assets, and good reputation. Treatment quality was the most important factor in mild illness with trusted service or medical personnel (loading=0.56) and good medical equipment (loading=0.42) as the two significant positive contributing factors. To receive the benefit of these positive contributing factors, residents were willing to neglect the cost of getting treatment (loading=-0.47) and distance from home (loading=-0.39). The major preference factors for chronic illness represented 59.2% variance (*n*=215). The major preference factors, from most important to less so, were: treatment quality, accessibility and acceptability, good communication, affordability and quality of life improvement. In the case of serious illnesses, 61.5% of the variance from 209 residents were represented by the major preference factors. Accessibility and acceptability were the most important factors for serious illness, followed by treatment quality, good reputation, affordability and high demand.

**Table 3:** Major preference factors in choosing primary care provider

**Variables**	**Variable loadings for Principal Components (PC)**					
	**Treatment quality (PC1)**	**Affordability (PC2)**	**Physical assets (PC3)**		**Good reputation (PC4)**	**Accessibility & acceptability (PC5)**
1. Mild illnesses (cumulative percentage=55.72%, *n*=211)						
Distance from home	-0.3947	-0.0688	0.3493		0.0940	0.5986
Waiting time	-0.0064	-0.5836	-0.0849		0.3933	-0.0368
Hospitality	0.2070	0.4468	-0.2581		0.3268	0.3513
Environment	0.3931	-0.0016	0.5722		0.2881	-0.3837
Involved in decision making	0.3853	0.3453	0.2437		0.0985	0.4939
Improvement after first visit or positive experience	0.3646	0.2848	-0.4644		0.0296	-0.0548
Cost to get treatment	-0.4679	0.4952	0.0030		-0.1503	-0.1771
Eligibility of using BPJS-Health or other insurance	-0.3750	0.5572	0.2333		-0.1103	-0.2929
Good medical equipment	0.4164	0.0785	0.3825		0.2729	-0.1710
Personal preference	0.5569	-0.1842	-0.1876		-0.5684	0.0090
Trusted service and or medical personnel	0.5602	0.2692	0.1350		-0.1771	0.0313
Recommendation from family or relatives	0.0007	0.1982	-0.4717		0.5324	-0.1779
**Variables**	**Variable loadings for Principal Components (PC)**					
	**Treatment quality (PC1)**	**Accessibility & acceptability (PC2)**	**Good communication (PC3)**		**Affordability (PC4)**	**Quality of life improvement (PC5)**
2.Chronic illnesses (cumulative percentage=59.22%, *n*=215)						
Distance from home	-0.4292	0.3532	-0.1399		-0.3872	-0.1954
Waiting time	0.0853	0.6068	-0.3161		0.0045	-0.3733
Hospitality	0.0732	0.6403	0.3555		0.2801	0.0935
Environment	0.4007	0.3991	0.3207		0.3713	-0.3545
Involved in decision making	0.1559	0.2092	0.4590		-0.4274	0.2170
Improvement after first visit or positive experience	0.0695	0.3194	0.2633		-0.1122	0.6600
Cost to get treatment	-0.6252	-0.1382	0.1334		0.3877	0.0410
Eligibility of using BPJS-Health or other insurance	-0.5571	-0.0800	0.3199		0.5213	-0.0198
Good medical equipment	0.6116	-0.2227	0.3425		0.0054	-0.2731
Personal preference	0.3658	0.0635	-0.3890		0.3548	0.4835
Trusted service and or medical personnel	0.5872	-0.3961	0.0666		0.1352	-0.0255
Recommendation from family or relatives	0.2290	0.2307	-0.5332		0.2568	0.1014
**Variables**	**Variable loadings for Principal Components (PC)**					
	**Accessibility & acceptability (PC1)**	**Treatment quality (PC2)**		**Good reputation (PC3)**	**Affordability (PC4)**	**High demand (PC5)**
3. Serious illnesses (cumulative percentage=61.45%, *n*=209)						
Distance from home	0.4448	-0.4779		0.2332	-0.3105	-0.1540
Waiting time	0.4746	0.1239		-0.4544	-0.0423	-0.3813
Hospitality	0.6686	0.2745		-0.1522	0.1024	0.0138
Environment	0.7563	0.3516		-0.0876	0.1077	0.0806
Involved in decision making	0.0065	0.1521		0.1747	-0.5626	0.6179
Improvement after first visit or positive experience	0.3957	0.3066		-0.1558	-0.1608	0.4515
Cost to get treatment	0.0099	-0.4774		-0.1716	0.4552	0.4414
Eligibility of using BPJS-Health or other insurance	0.2693	-0.4482		0.0490	0.4981	0.2859
Good medical equipment	-0.2468	0.6593		-0.0855	0.1422	0.0582
Personal preference	0.0686	0.2330		0.7378	0.1837	-0.0805
Trusted service and or medical personnel	-0.3511	0.6263		0.0230	0.3338	0.0637
Recommendation from family or relatives	0.5236	0.1414		0.5067	0.1461	-0.0812

*BPJS-Health* Badan Penyelenggara Jaminan Sosial Kesehatan (Indonesian National Health Insurance)

### Major preference factors associated with primary care choice

Table 4 shows major preference factors that were associated with primary care choice in mild illnesses. Treatment quality factor was associated with the willingness to choose self-medication (RRR = 2.44; 95% CI, 1.44-4.14, *P* = 0.001) and non-BPJS-Health partners or hospital (RRR = 4.70; 95% CI, 2.44-9.03, *P* = <0.001), compared to BPJS-Health partners. Residents preferred to choose a pharmacy other than BPJS-Health partners because of accessibility and acceptability (RRR = 6.77; 95% CI, 2.37-19.32, *P* = <0.001) and affordability (RRR = 0.23; 95% CI, 0.11-0.49, *P* = <0.001). However, a significant association was found between choosing affordability as an important factor and choosing BPJS-Health partners, compared to all other primary care choices. In chronic (RRR self-medication=3.26; non-BPJS-Health=11.97) and serious (AOR non-BPJS-Health=6.84; 95% CI, 3.62-12.92, *P* = <0.001) illnesses, treatment quality remained a factor that was negatively associated with the decision to choose BPJS-Health partners.

**Table 4:** Major preference factors associated with primary care choice

**Condition**	**Variables**	**Primary care choice (AOR/RRR [95% CI])**		
		**Pharmacy**	**Self-medication**	**Non-BPJS-Health**
Mild illnesses (*n*=211)	Treatment quality	1.53 (0.55-4.26)	2.44 (1.44-4.14)**	4.70 (2.44-9.03)***
	Affordability	0.23 (0.11-0.49)***	0.36 (0.23-0.56)***	0.32 (0.17-0.58)***
	Physical assets	0.54 (0.16-1.80)	0.86 (0.50-1.47)	1.03 (0.59-1.79)
	Good reputation	0.60 (0.24-1.47)	0.94 (0.59-1.50)	2.12 (1.25-3.60)*
	Accessibility and acceptability	6.77 (2.37-19.32)***	1.22 (0.77-1.96)	1.38 (0.78-2.43)
Chronic Illnesses (*n*=214)	Treatment quality		3.26 (1.41-7.51)*	11.97 (6.28-22.81)***
	Accessibility and acceptability		0.86 (0.42-1.75)	1.06 (0.71-1.59)
	Good communication		0.26 (0.12-0.53)	0.73 (0.45-1.17)
	Affordability		0.82 (0.44-1.54)	0.83 (0.56-1.24)
	Quality of life improvement		1.06 (0.85-2.76)	1.06 (0.70-1.61)
Serious Illnesses (*n*=201)	Accessibility and acceptability			1.35 (0.53-3.45)
	Treatment quality			6.84 (3.62-12.92)***
	Good reputation			0.62 (0.36-1.07)
	Affordability			0.91 (0.59-1.39)
	High demand			0.95 (0.52-1.71)

^*^p<0.05, **p<0.01, ***p<0.001

Reference category: BPJS-Health partners

*BPJS-Health* Badan Penyelenggara Jaminan Sosial Kesehatan (Indonesian National Health Insurance), *AOR* Adjusted odds ratio, *RRR* Relative risk ratio, *CI* Confidence interval

## Discussion

This study examined factors associated with previous and hypothetical settings of different primary care choices. PBI (*Penerima Bantuan Iuran*)—those who were fully covered by the government—was positively associated with more frequent use of BPJS-Health partners during the previous year. In contrast, being registered to other insurance was negatively associated with more frequent use of BPJS-Health partners. In addition to health insurance status, disease history, and socio-demographic characteristics were influencing residents' primary care choices in different severity of illnesses. Residents’ preference for the providers’ characteristics, such as treatment quality and affordability, was also associated with their choice of primary care provider.

In areas with high availability of non-BPJS-Health clinics, knowing the residents’ preference factor is important as an input for policymakers in deciding future BPJS-Health partnerships. Based on PCA analysis, treatment quality was found as the most important factor in choosing a primary care provider in mild and chronic illnesses. It was also the second major preference factor when having serious illnesses. However, the treatment quality factor was negatively associated with choosing BPJS-Health partners in all severity of illnesses. At the national level, only 67% of the puskesmas received a passing grade for preparedness in 2017 [23]. Low treatment quality of primary care providers could also be the reason for overwhelmed hospitals or so-called ‘giant puskesmas’ in urban areas, caused by the people who seek care for 155 illnesses that should be treated in the primary care level [24, 25].

Residents’ choice of primary care providers was influenced by insurance status and monthly household income. Being PBI was positively associated with more frequent use of BPJS-Health partners. It is consistent with the previous study which stated that the population with lower expenditure in urban areas was more likely to choose public health centers after the BPJS-Health implementation [26]. PBI preferred to choose BPJS-Health partners despite the perceived low treatment quality, even when having serious illnesses. In contrast, being registered to other insurance was negatively associated with frequent use of BPJS-Health partners. Moreover, having high income was negatively associated with frequent use of BPJS-Health partners. The affordability factor was positively associated with choosing BPJS-Health partners when having mild illnesses. BPJS-Health implementation has increased the affordability of health services [23]. Health insurance has increased the access to outpatient services in Indonesia and the PBI implementation increased the public health facilities utilization [26, 27]. However, PBI who were residents with low income (less than Rp 600,000 per month) could only access a limited number of BPJS-Health partners that perceived with poor treatment quality. When residents have a high monthly income and can pay for other insurance premiums, they can access a variety of health facilities.

Residents’ disease history affected residents’ choice of primary care services across the severity of illnesses. Among residents with chronic disease, they prefer to do self-medication unless they feel that the illness condition is serious. As the treatment quality factor was the most important in chronic illnesses condition, residents with chronic illnesses might prefer to do self-medication because of this issue. Primary care providers in developing countries tend not to be ready to provide even basic care for chronic illnesses [28–30]. Another study in Egypt showed that the presence of chronic disease is associated with self-medication practice with complementary or alternative medicine [31]. Complementary or alternative medicine is also culturally practiced in Indonesia for self-medication for many centuries [32].

This study has several limitations. First, when determining sampling areas using spatial analysis, the catchment areas were not created by considering the traffic condition. However, different speed rate from OpenStreetMap of each road type was applied as an approach to overcome this limitation. Another limitation was regarding the hypothetical type of question. This type of question might not reflect the real decision of the residents. The annual medical expenses were also asked verbally without any document to prove the actual amount. This might caused recall bias.

Despite those limitations, this study has some strengths as well. To the best of our knowledge, this is the first study to analyze primary care choice under health insurance schemes in specific areas determined by spatial network analysis. The findings from this study might be helpful for policymakers, especially regarding the future BPJS-Health partnership in low BPJS-Health partners coverage. The use of spatial analysis also adds the strength of the sampling method.

## Conclusions

Residents’ characteristics and preference factors all affect residents’ primary care choice in different types of illness severity. Residents with PBI status of BPJS-Health were more likely to get treatment from BPJS-Health partners. Affordability and treatment quality were two important factors in choosing or not to choose BPJS-Health partners.

By spatial analysis, the beneficiaries’ position of a household can be linked with BPJS-Health partners’ catchment area and it will make the redistribution process much easier. Furthermore, if the redistribution process is not enough to achieve the target ratio of 1 : 5000, spatial analysis can also be used for analyzing potential non-BPJS-Health partners to be recruited as BPJS-Health partners. If some non-BPJS-Health partners are available in one area, as described in the sampling area of this study, the preference factors could be used to select the potential provider for the partnership.

### Supplementary Information

**Additional file 1.** Questionnaire (English Version).

## Data Availability

The datasets generated and analysed during the current study are not publicly available due to ethical reason but are available from the corresponding author on reasonable request.

## Abbreviations

BPJS-Health: *Badan Penyelenggara Jaminan Sosial Kesehatan* (Indonesian national health insurance)
LMICs: Low-income and middle-income countries
Puskesmas: *Pusat kesehatan masyarakat* (government primary health centres)
PCA: Principal component analysis
PBI: *Penerima Bantuan Iuran* (Full-covered BPJS-Health beneficiaries by government)
AOR: Adjusted odds ratio
RRR: Relative risk ratio
