# Supplementary material for: What are the factors affecting primary care choice when the access under health insurance scheme is limited?: a cross-sectional study in Bandung, Indonesia
Source: BMC Prim Care. 2024 Feb 21;25:64. doi: 10.1186/s12875-024-02296-6 (PMC10882734; doi:10.1186/s12875-024-02296-6)
Supplement: Supplementary file 1 — Additional file 1. Questionnaire (English Version). [file 12875_2024_2296_MOESM1_ESM.pdf]

Questionnaire (English Version)

What are the factors affecting primary care choice when the access under insurance scheme is limited? : a cross-sectional study in Bandung, Indonesia

1. Health Service Utilization

S.N.	Questions	Answers	
101	How do you feel about your health condition?	1. Very poor	
		2. Poor	
		3. Moderate	
		4. Well	
		5. Very well	
102	Do you have chronic disease or other health issues that needs monitoring?	1. Yes	
		2. No	
103	In the past year, did you have any (other) health issues? (acute illnesses, minor ailments such as fever, cough, cold, diarrhea, dizziness, etc.)	1. Yes	
		2. No	
104	In the past year, how many times did you consult with doctors?	1. 0	
		2. 1-3 times	
		3. >3 times	
105	In the past year, have you ever hospitalized?	1. Yes	
		2. No	
106	How much is your annual medical expenses?	1. <Rp 250.000,00	
		2. Rp 250.000,00 - Rp 2.000.000,00	
		3. Rp 2.000.000,00 - Rp 5.000.000,00	
		4. Rp 5.000.000,00	
107	How do you feel about the medical cost?	1. Can not undertake	
		2. Can mainly undertake	
		3. Can entirely undertake	
108	Which BPJS-Health beneficiaries are you?	1. PBI (under government support)	
		2. Non PBI	
109	Do you have any other health insurance other than BPJS-Health?	1. No	
		2. Private health insurance	
		3. Insurance from company/office	
110	When do you use BPJS-Health benefit? (check the box)	□ Mild illnesses (check list no.1)	
		□ Chronic illnesses (check list no.2)	
		□ Serious illnesses (other than in check list)	
111	If you have other health insurance, when do you use its benefit?	□ Mild illnesses (check list no.1)	
		□ Chronic illnesses (check list no.2)	
		□ Serious illnesses (other than in check list)	

2. Preference of Health Care

S.N.	Questions	Answers	
201	If you have mild illnesses (see list no. 1), will you seek care from any provider?	1. No (go to 202)	
		2. Yes (go to 203)	
202	What kind of action will you do for mild illnesses?	1. Do nothing	
		2. Self-treatment (home remedy)	
		3. Go to drug store or using available drugs at home	
		4. Buy drug directly from community pharmacy	
203	Which provider will you choose to seek for care in mild illnesses?	1. Community pharmacy (consult with pharmacist)	
		2. *Puskesmas/pustu* or UKBM (*poskesdes, plindes posyandu* or *balai pengobatan*)	
		3. Private clinic/physician (BPJS-Health partner)	
		4. Private clinic/physician/midwife (non BPJS-Health partner)	
		5. Hospital	
		6. Others (please specify)	
204	What are the important factors to choose or not to choose the health care provider in mild illnesses?	□ Waiting time	
		□ Hospitality	
		□ Good environment	
		□ Involved in decision making	
		□ Improvement after first visit	
		□ Low cost to get treatment	
		□ Eligiblity of using BPJS-Health/other insurance	
		□ Good medical equipment	
		□ Personal preference	
		□ Previous positive experience	
		□ Recommendation from family	
		□	
205	If you have chronic illnesses (see list no. 2), will you seek care from any provider?	1. No (go to 206)	
		2. Yes (go to 207)	
206	What kind of action will you do for chronic illnesses?	1. Do nothing	
		2. Self-treatment (home remedy)	
		3. Go to drug store or using available drugs at home	
		4. Buy drug directly from community pharmacy	
207	Which provider will you choose to seek for care in chronic illnesses?	1. Community pharmacy (consult with pharmacist)	
		2. *Puskesmas/pustu* or UKBM (*poskesdes, plindes posyandu* or *balai pengobatan*)	

		3. Private clinic/physician (BPJS-Health partner)	
		4. Private clinic/physician/midwife (non BPJS-Health partner)	
		5. Hospital	
		6. Others (please specify)	
208	What are the important factors to choose or not to choose the health care provider in chronic illnesses?	1. Waiting time	
		2. Hospitality	
		3. Good environment	
		4. Involved in decision making	
		5. Improvement after 1st visit	
		6. Low cost to get treatment	
		7. Eligiblity of using BPJS-Health/other insurance	
		8. Good medical equipment	
		9. Personal preference	
		10. Previous positive experience	
		11. Recommendation from family	
209	If you have serious illnesses or other health issues (not mentioned in list no. 1 and 2.), will you seek care from any provider?	1. No (go to 210)	
		2. Yes (go to 211)	
210	What kind of action will you do for serious illnesses or other health issues (not mentioned in list no. 1 and 2.)?	1. Do nothing	
		2. Self-treatment (home remedy)	
		3. Go to drug store or using available drugs at home	
		4. Buy drug directly from community pharmacy	
211	Which provider will you choose to seek for care in serious illnesses or other health issues (not mentioned in list no. 1 and 2.)?	1. Community pharmacy (consult with pharmacist)	
		2. *Puskesmas/pustu* or UKBM (*poskesdes, plindes posyandu* or *balai pengobatan*)	
		3. Private clinic/physician (BPJS-Health partner)	
		4. Private clinic/physician/midwife (non BPJS-Health partner)	
		5. Hospital	
		6. Others (please specify)	
212	What are the important factors to choose or not to choose the health care provider in serious illnesses or other health issues (not mentioned in list no. 1 and 2.)?	1. Waiting time	
		2. Hospitality	
		3. Good environment	
		4. Involved in decision making	
		5. Improvement after 1st visit	
		6. Low cost to get treatment	

		7. Eligiblity of using BPJS-Health/other insurance	
		8. Good medical equipment	
		9. Personal preference	
		10. Previous positive experience	
		11. Recommendation from family	

3. Socio-demographic Information

S.N.	Questions	Answers	
301	What is your relationship with household holder?	1. Household holder	
		2. Wife/husband	
		3. Child/step child	
		4. Daughter/son in-laws	
		5. Grandchild	
		6. Parents/in-laws	
		7. Others (no family relationship)	
302	What is your marrital status?	1. Single	
		2. Married	
		3. Divorce/widowed	
303	What is your gender?	1. Male	
		2. Female	
304	What is your completed age (in years)?		
305	What is your highest level of education?	1. Illiterate	
		2. Can read and write only	
		3. Elementary school (until grade 6 or equal)	
		4. Junior high school (until grade 9 or equal)	
		5. Senior high school (until grade 12 or equal)	
		6. Diploma 1, Diploma 2 or equal	
		7. Diploma 3	
		8. Bachelor	
		9. Masters	
		10. Masters and above	
306	What is your main occupation?	1. Entrepreneur	
		2. Freelancer	
		3. Civil servant	
		4. Office worker/employee	
		5. Family worker/housewife/no income	
		6. Retiree	
		7. Student	
		8. Others (please specify)	

307	What is household holder highest level of education?	1. Illiterate	
		2. Can read and write only	
		3. Elementary school (until grade 6 or equal)	
		4. Junior high school (until grade 9 or equal)	
		5. Senior high school (until grade 12 or equal)	
		6. Diploma 1, Diploma 2 or equal	
		7. Diploma 3	
		8. Bachelor	
		9. Masters	
		10. Masters and above	
308	What is household holder main occupation?	1. Entrepreneur	
		2. Freelancer	
		3. Civil servant	
		4. Office worker/employee	
		5. Family worker/housewife/no income	
		6. Retiree	
		7. Student	
		8. Others (please specify)	
309	How much is your family income per month?	1. Less than Rp 600.000,00	
		2. Rp 600.000,00 - Rp 5.000.000,00	
		3. Rp 5.000.000,00 - Rp 15.000.000,00	
		4. More than Rp 15.000.000,00	
